# Retrospective study on factors affecting the prognosis in oral cancer patients who underwent surgical treatment only

**DOI:** 10.1186/s40902-015-0047-8

**Published:** 2016-01-16

**Authors:** Byeong-Guk Kim, Jun-Hwa Kim, Myung-In Kim, Jeong Joon Han, Seunggon Jung, Min-Suk Kook, Hong-Ju Park, Sun-Youl Ryu, Hee-Kyun Oh

**Affiliations:** Department of Oral and Maxillofacial Surgery, School of Dentistry, Dental Science Research Institute, Chonnam National University, 77, Yongbongro, Buk-Gu, Gwangju, 500-757 South Korea

**Keywords:** Neoplasm metastasis, Oral cancer, Recurrence, Survival rate, TNM classification

## Abstract

**Background:**

This study was performed to evaluate their 5-year survival rates and identify the factors affecting the prognosis of oral cancer patients who had undergone surgical treatment only.

**Methods:**

Among 130 patients who were diagnosed with malignant tumor of oral, maxillofacial, and surgical treated in the Department of Oral and Maxillofacial Surgery at Chonnam National University Hospital within a period from January 2000 to December 2010, for 11 years, 84 patients were investigated who were followed up for more than 5 years after radical surgery; oral cancer is primary and received only surgical treatment. The survival rate according to gender, age, type and site of cancer, TNM stage, cervical lymph node metastasis and its stage, recurrence or metastasis, time of recurrence and metastasis, and differentiation were investigated and analyzed.

**Results:**

Overall, 5-year survival rate in patients who received only surgical treatment was 81.2 %, and disease-specific 5-year survival rate was 83.1 %. The disease-specific 5-year survival rate based on TNM stage, metastasis of cervical lymph node, N stage, and presence of recurrence/metastasis was a significant difference (*p* < 0.05). The disease-specific 5-year survival rate based on sex, age, type of tumor, primary site, and differentiation was not a significant difference (*p* > 0.05).

**Conclusions:**

These results suggest that good survival rate can be obtained with surgical treatment only, and stage of oral cancer, cervical lymph node metastasis and stage, recurrence or metastasis, time of recurrence, and metastasis have a significant effect on survival rate in oral cancer patients.

## Background

For the treatment of oral cancer, it is still controversial, but usually, surgical treatment is preferred in the initial oral cancer and the cases of progressed oral cancer like cervical lymph node metastasis or extracapsular spread have been performed with surgical treatment along with combination therapy and radiation therapy [1]. Recently, it has emerged as an important factor of treatment decisions, quality of life in addition to the possibility of oral cancer recovery [2].

Radiation therapy and chemotherapy in patients with oral cancer performed separately and also performed before surgery or after surgery. Radiation therapy may be used for tongue cancer effectively but is performed limitedly because of the influence on the adjacent normal tissues [3]. It induces side effects such as induction of malignant neoplasm, osteoradionecrosis (ORN), pronunciation disorders, dysphagia, dry mouth, and dental caries [4]. In addition, it has limited radiation therapy to perform radiation therapy again in the same site and it makes more complicated that there is a salvage treatment through surgery after radiotherapy [3]. Chemotherapy is applied to advanced stage, extracapsular spread, recurrence or metastasis, and the case of palliative therapy.

It is reported that the prognosis of oral cancer patients undergoing radiation therapy or combination therapy after surgical treatment is not significantly better than those who received only surgical treatment [4]. Performing only surgical treatment is preferred since it prevents the side effects of chemotherapy and radiation therapy and obtains a good result. Wolfensberger et al. reported the disease-specific 4-year survival rate of 94 % and recurrence or metastasis rate of 18 % in the 93 cases only through surgical procedures in oral cancer patients, and Lim et al. reported the disease-specific 5-year survival rate of 83 % and recurrence or metastasis rate of 21 % in the 76 cases [4, 5]. Liu et al. reported a 5-year survival rate of 72 % and recurrence or metastasis rate of 25 % in 72 cases [6].

There are a few reports on the factors that affect the prognosis of oral cancer patients. Massano et al. reported that TNM stage, extracapsular spread, resection margins of lesions, and the thickness of the tumor have high relevance to the prognosis of oral squamous carcinoma patients; Rajapakshe et al. reported that factors which affect the prognosis and survival of oral squamous carcinoma patients are TNM staging, lymph node metastasis, and the status of the resection margin of lesions [7–9].

This study was performed to evaluate their 5-year survival rates and identify the factors affecting the prognosis of oral cancer patients who had undergone surgical treatment only.

## Methods

1. Patients
  - Among oral cancer patients who have received the surgical treatment in the Department of Oral and Maxillofacial Surgery at Chonnam National University Hospital within a period from January 2000 to December 2010, for 11 years, 84 patients were investigated who were followed up for more than 5 years, had primary oral cancer, and received only surgical treatment.
2. Methods
  - Examination of patients’ medical records
    The patient’s medical records were examined. The clinical, pathological, and medical care information were collected retrospectively. Biopsy for diagnosis, computed tomography (CT), whole body bone scan (WBBS), and positron emission computed tomography-computed tomography (PET-CT) findings of such were examined. Overall survival rates, etc. were investigated after categorization referring to the classification table (AJCC cancer staging manual 7th edition) recommended by AJCC for the distribution of the survival status and location of the oral cavity of the patient [10].
  - Surgical treatment
    Patients with tumor-free dissection boundaries and who cannot get radiation treatment for cancer underwent surgical treatment. Radical resection was performed including a safety margin of 10 mm. Neck dissection was performed in 82 patients among 84 patients, bilateral supraomohyoid neck dissection (SOHND) in 75 patients, ipsilateral SOHND in 5 patients, ipsilateral SOHND and opposite SND (Selective neck dissection: level I only) in a patient, and bilateral modified radical neck dissection (MRND) in a patient. Direct closure was performed in 46 cases (55 %) of 84 cases and reconstruction in 38 cases (45 %). Local flap (16 %) for reconstruction was in 6 cases, and microvascular free flap was performed in 32 cases (84 %).
  - Prognosis assessment of patients
    The 5-year survival rate and the disease-specific 5-year survival rate were calculated. Factors affecting the prognosis of oral cancer patients, including gender and age of patients, type, stage and location of cancer, lymph node metastasis, stage of lymph node, recurrence and metastasis, time of recurrence and metastasis, and differentiation, were investigated.
    Standard of classification table (AJCC cancer staging manual 7th edition) recommended by the AJCC is applied to TNM classification for staging of cancer [10].
  - Statistical assessment
    The survival rate was calculated using the Kaplan Meier method, and the log rank test was performed for significance test of the predicted factors that affect the prognosis. Each analysis was performed using the SPSS 20 (IBM, Chicago, Illinois, USA).

## Results

1. Prognosis according to gender and age
  Oral cancer patients receiving surgery treatment were 58 male and 26 female, and the rate was higher 2.3 times in men, and overall, 5-year survival rate was 81.2 %. The disease-specific 5-year survival rate according to gender (women 84.6 %, men 82.5 %) showed no significant difference, and results of log rank test showed that sex does not affect prognosis (Table 1).

**Table 1:** Vital status and disease specific 5-year survival rate according to gender

Gender	No. of cases	Alive			Dead			DSS
		TFNR	TFAR	AWPRM	DTF	DWC	DPO	
Male	58	38	5	4	4	5	2	82.5
Female	26	17	2	2	2	2	1	84.6
Total	84	55	7	6	6	7	3	83.1

*ATFNR* alive; tumor-free; no recurrence, *ATFAR* alive; tumor-free; after recurrence, AWPRM alive; with persistent/recurrent/metastatic disease, *DTF* dead; tumor-free, *DWC* dead; with cancer-primary/recurrent/metastatic, *DPO* dead; postoperative, *DSS* disease specific 5-year survival rate
  According to the age distribution of oral cancer patients, affected age was 60 or more (55 patients, 65 %). Disease specific 5-year survival rate by age decreased slightly with age, but there was not statistically significant difference, and the result of log rank test showed that age does not affect the prognosis (Fig. 1).

**Fig. 1:**
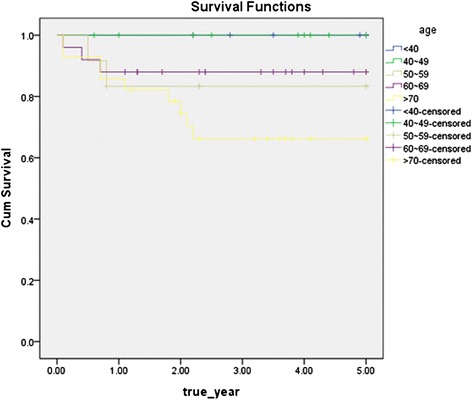
Disease specific 5-year survival rate by age group (*p* = 0.093)
2. Prognosis according to type, site, and stage of cancer
  Squamous cell carcinoma among oral cancer was the most common with 62 cases (71.2 %); the disease-specific 5-year survival rate of these patients was 82.0 %. The disease-specific 5-year survival rate according to the type of oral cancer ranged from 100 to 0 %. Results of Kaplan Meier method and log rank test showed that the type of oral cancer does not affect prognosis (Table 2).

**Table 2:** Disease specific 5-year survival rate according to type of neoplasm

Type	No. of cases (%)	Recur or meta	DSS
SCC	62 (71.2)	15	82
ACC	7 (8.2)	1	100
MM	4 (4.7)	1	75
VC	4 (4.7)	0	100
Sar	3 (3.5)	1	66.6
MC	2 (2.3)	1	100
BCC	1 (1.8)	0	100
GCC	1 (1.8)	0	100
Total	84 (100.0)	18	83.1

*SCC* squamous cell carcinoma, *ACC* adenoid cystic carcinoma, *MM* malignant melanoma, *VC* verrucous carcinoma, *Sar* sarcoma, *MC* mucoepidernoid carcinoma, *BCC* basal cell carcinoma, *GCC* ghost cell carcinoma, *Recur* recurrence, *meta* metastasis, *DSS* disease specific 5-year survival rate)
  Areas most affected by oral cancer were anterior two-thirds of the tongue followed by floor of the mouth, inferior alveolar ridge. The disease-specific 5-year survival rate with the site of oral cancer ranged from 100.0 to 69.2 %, and results of log rank test showed that site of oral cancer does not affect prognosis (Table 3).

**Table 3:** Disease specific 5-year survival rate according to primary site of neoplasm

Primary site	No. of cases (%)	Recur or meta	DSS
Tongue	18 (21.4)	4	88.9
FOM	14 (16.7)	4	76.9
LAR	13 (15.5)	3	69.2
HP	9 (10.7)	1	87.5
ML	9 (10.7)	1	100
RT	7 (8.3)	3	71.4
SP	6 (7.1)	0	83.3
BM	5 (6.0)	1	100
UAR	3 (3.6)	1	100
Total	84 (100.0)	18	83.1

*Tongue* anterior 2/3 of the tongue, *FOM* floor of mouth, *LAR* lower alveolar ridge, *HP* hard palate, *ML* mucosal lip, *RT* retromolar trigone, *SP* soft palate, *BM* buccal mucosa, *UAR* upper alveolar ridge
  In the stage of patients, 25 people belong to stage I, 23 people to stage II, 16 people to stage III, and 20 people to stage IV, as stage progresses, the disease-specific 5-year survival rate decreases as follows: 96.0 % of patients belong to stage I, 90.9 % of patients to stage II, 86.7 % of patients to stage III, 57.1 % of patients to stage IV, and the *p* value is 0.003 by log rank test results, which showed the stage was significant factor in the survival rate (Fig 2).

**Fig. 2:**
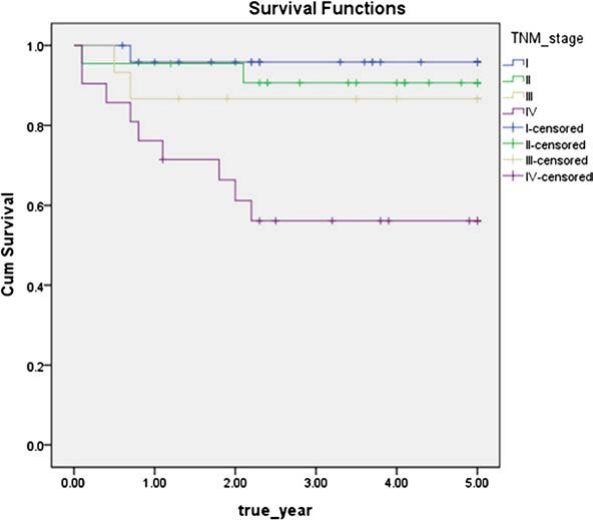
Disease specific 5-year survival rate by TNM stage (*p* = 0.003)
3. Prognosis according to metastasis of cervical lymph node and stage of cervical lymph node
  The disease-specific 5-year survival rate without cervical lymph node metastasis was significantly higher than that with cervical lymph node metastasis (93.2 vs 58.3 % (Fig 3)). As N stage progressed, the disease-specific 5-year survival rate significantly decreased (*p* < 0.05) (Fig 4).

**Fig. 3:**
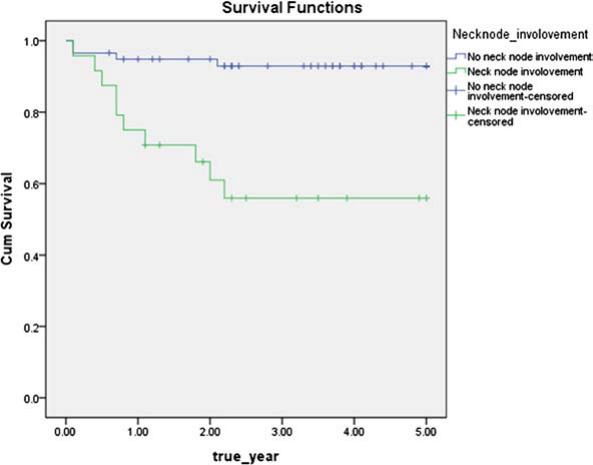
Disease specific 5-year survival rate by positive neck node (*p* = 0.000)

**Fig. 4:**
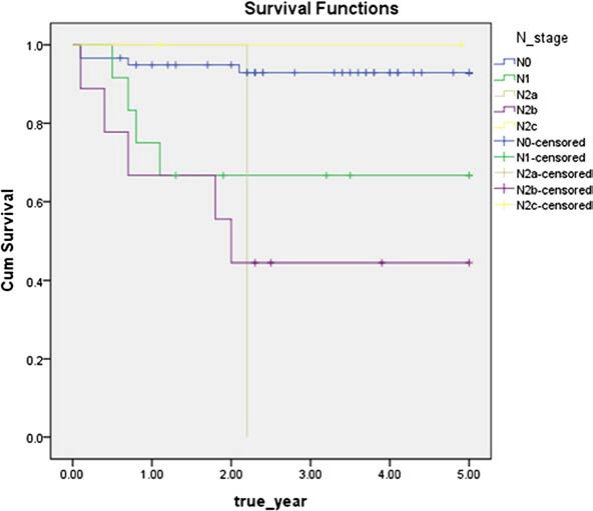
Disease specific 5-year survival rate by N stage (*p* = 0.000)
4. Prognosis according to recurrence/metastasis or timing of recurrence/metastasis
  The disease-specific 5-year survival rate according to recurrence or metastasis recurrence or metastasis was 89.1 % in the case without recurrence or metastasis and 63.2 % with recurrence or metastasis, and whether or not, recurrence and metastasis were significant factors, since significant probability was 0.011 by log rank test results, which showed that recurrence and metastasis were significant factors in the survival rate (Fig 5). The survival rate varied according to the time of recurrence or metastasis of 19 patients who experienced postoperative recurrence or metastasis. The disease-specific 5-year survival rate of patients who experienced recurrence or metastasis within 1 year after surgery was 45.5 %, within 1–2 years was 85.7 %, and within 2–3 years was 100 %, and significant probability was 0.002 by log rank test results, which showed that the time of recurrence or metastasis after surgery was a significant factor in the survival rate (Fig 6).

**Fig. 5:**
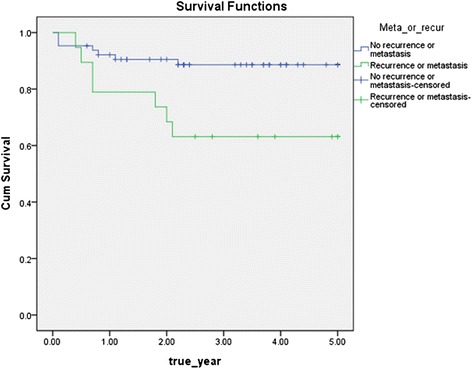
Disease specific 5-year survival rate by recurrence / metastasis (*p* = 0.011)

**Fig. 6:**
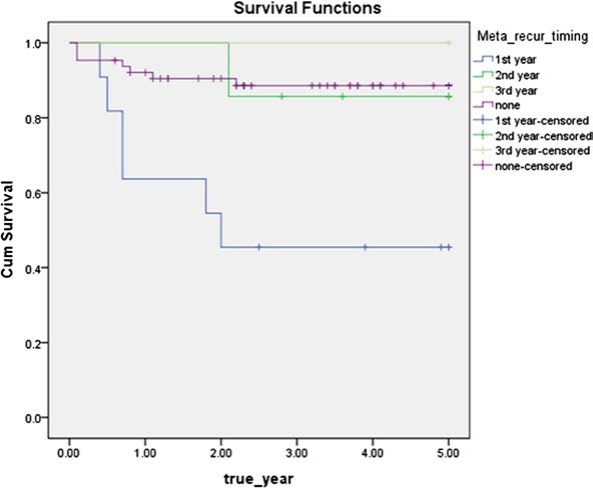
Disease specific 5-year survival rate by timing of recurrence/metastasis (*p* = 0.002)
  Recurrence or metastasis occurs to all 18 patients (21.4 % of 84 patients), with local recurrence only occurring to 7 patients, regional recurrence only to 8 patients, local recurrence and regional recurrence to 1 patient, and with distant metastasis to 2 patients. One of 7 patients has local recurrence only, 3 of 8 patients with regional recurrence only, one patient with locoregional recurrence, and 1 of 2 patients with distant metastasis died (Table 4). It was most common that recurrence or metastasis occurred within 1 year to 10 (58 % of 18 patients) of 18 cases, within 1–2 years to seven cases (37 % of the 18 patients), within 2–3 years to one case (5 % of 18 patients), and most recurrence or metastasis occurred within 2 years (95 %) after surgery.

**Table 4:** The number of cases and death of the patients who had recurrence or meta

Recur or meta	No. of cases (%)	No. of death
Local recur only	7 (38.9)	1
Regional recur only	8 (44.4)	3
Locoregional recur	1 (55.6)	1
Distant meta	2 (11.1)	1
Total	18 (100.0)	6

**Fig. 7:**
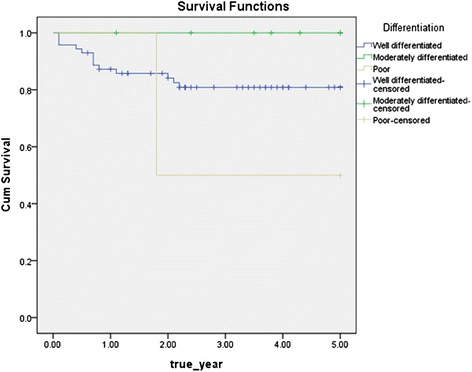
Disease specific 5-year survival rate by histopathological differentiation (*p* = 0.189)
  Out of the 7 patients with local recurrence, 5 patients were T1-2 stage and 2 patients were T3-4 stage. Categorizing according to differentiation, well differentiated type was 5 % (4 of 73 patients) and moderately differentiated type was 30 % (3 out of 10 patients).

*Recur* recurrence, *meta* metastasis, *DSS* disease specific 5-year survival rate
5. Prognosis according to differentiation
  Depending on the histopathological differentiation, the disease specific 5-year survival rate was 81.7 % (58 out of 73 patients survival) for the well differentiated type, 100.0 % (10 out of 10 patients survival) for the moderately differentiated type, and 100.0 % (1 out of 1 patients survival) for the poorly differentiated type, and *p* value was 0.189 by log rank test results, which showed that the histopathological differentiation was not a significant factor in the survival rate (Fig 7).

## Discussion

Radiotherapy or chemotherapy after the surgical procedure is largely determined by the histopathological findings of the lesion in the treatment decision of oral cancer [11, 12]. According to Brown et al., who reported that the overall 5-year survival rate of surgical treatment and surgical treatment accompanied by postoperative radiotherapy was 71 and 54 %, respectively, for 193 patients with oral squamous cell carcinoma of TNM stage I-II, good result can be obtained only through surgical treatment in the clean resection boundaries and the lesion of low stage (Stage I-II) with low recurrence probability [13]. In this study, the effect that sex, age, type of oral cancer, primary site, stage, cervical metastasis, stage of lymph node, metastasis depending on neck level, recurrence or metastasis, time of recurrence or metastasis, etc. has on survival rate was evaluated.

There are reports that showed excellent prognosis only through surgical treatment in oral cancer patients. Lim et al. reported a disease-specific 5-year survival rate of 83 % with only performing surgical procedure in 76 oral cancer patients [4]. Magge et al. reported that the prognosis of surgical treatment accompanied by postoperative radiotherapy compared to only surgical treatment did not improve [9]. In this study, disease-specific 5-year survival rate was 83.1 % only through surgical treatment for the oral cancer patients. This is similar when compared to the results reported in the other literature so far [14, 15].

In this study, with the gender distribution of oral cancer patients about 2.3:1 ratio (58 male, 26 female), the proportion of men was higher. The result was similar to gender distribution of the literature researched in Korea [4, 16]. There was no significant difference in the disease-specific 5-year survival rate by gender (male 82.5 %, female 84.6 %) as other reports (Liu et al., Roger et al.) [6, 17].

The effect that the age of oral cancer patients with surgical treatment has on prognosis has been controversial. Rogers et al. reported that as the age of the patient increase, disease-specific 5-year survival rate decreases, but Liu et al. reported that there was no significant differences statistically [6, 17]. In this study, year survival rate was slightly lower in the elderly after 50, but there was no significant difference.

With the result that squamous cell carcinoma patients only got surgical treatment, Lim et al. reported that 5-year survival rate was 83 % out of 76 patients, and Liu et al. reported that 5-year survival rate was 77 % out of 72 patients [4, 6]. In this study, the disease-specific 5-year survival rate was 82.0 % in the squamous cell carcinoma, melanoma, and sarcoma compared to the other tumor that showed a slightly lower survival rate, so the result was similar to the other literature [18–20], which was not statistically significant. The disease-specific 5-year survival rate based on type of tumor was not a significant difference.

Shah et al. reported that oral cancer showed another biological aspect according to primary site [21]. On the other hand, carcinomas on mucosal lip showed a good prognosis; carcinomas on anterior 2/3 of the tongue, floor of the mouth, and the lower alveolar ridge have high risk of metastasis to adjacent lymph nodes and showed a relatively poor prognosis. Rogers et al. reported that the disease-specific 5-year survival rate depending on primary site was 64–44 %, which was not statistically significant in the 489 oral cancer patients [17]. In this study, the disease-specific 5-year survival rate depending on primary site varied from 100.0 to 69.2 %, and there was no significant difference by the log rank test results.

Rajapakshe et al. and Geum et al. reported that TNM stage is the factor that has significant influence on the prognosis of oral cancer patients [8, 22]. In this study, as the stage increases, the disease-specific 5-year survival rate decreases (*p* = 0.003).

With the result only treated surgically for the 489 oral cancer patients, Rogers et al. reported that the disease specific 5-year survival rate (87 %) of the case without cervical lymph node metastasis was significantly higher than that of the case (54 %) with cervical lymph node metastasis [17]. In this study, the disease specific 5-year survival rate of the case (93.2 %) without cervical lymph node metastasis was significantly higher than that of the case (58.3 %) with cervical lymph node metastasis, which accorded with previous researches [4, 6, 17].

In study of Rogers et al., the disease-specific 5-year survival rate of N0, N1, and N2-3 stage was 87, 68, and 40 %, respectively [17]. In this study, the disease-specific 5-year survival rate according to cervical lymph node stage was 93.2 % for the N0 (60 patients), 66.7 % for the N1 (13 patients), 0 % for the N2a (a patient), 50.0 % for the N2b (8 patients), 100.0 % for the N2c (2 patients), and by the log rank test results, cervical lymph node stage had significant effects on oral cancer prognosis (*p* = 0.000).

Geum et al. reported that the disease-specific 5-year survival rate was 90.5 % for the patients without recurrence or metastasis and 30.0 % for the patients with recurrence or metastasis out of 37 oral cancer patients [22]. In this study, the disease-specific 5-year survival rate depending on recurrence or metastasis was 89.1 % for the case without recurrence or metastasis, was 63.2 % for the case with recurrence or metastasis, and by the log rank test results, recurrence or metastasis had an significant impact on oral cancer prognosis (*p* = 0.011).

Liu et al. reported that 72.2 % experienced a recurrence or metastasis after surgery within 2 years and 100 % did within 3 years out of patients with recurrence or metastasis [6]. In this study, 95 % experienced a recurrence or metastasis after surgery within 2 years and 100 % did within 3 years out of 18 cases with recurrence and metastasis, which was similar to report by Liu et al. [6].

Schwartz et al. reported that survival rate and prognosis of patients who experienced recurrence or metastasis after 6 months of primary operation were satisfactory than those of within 6 months in the study for 350 oral squamous cell carcinoma patients [23]. The disease-specific 5-year survival rate of those who experienced recurrence or metastasis within 1 year after surgery was 45.5 %, within 1~2 years was 85.7 %, and within 2~3 years was 100 %. As the recurrence or metastasis occurred early, prognosis was significantly poor, in results of the log rank test (*p* = 0.002).

Geum et al. reported that the disease-specific 5-year survival rate was 94.7 % for the well-differentiated type, 57.1 % for the moderately differentiated type, and 25.0 % for the poorly differentiated type related to survival rate of oral squamous cell carcinoma according to histopathological differentiation [22]. But Liu et al. reported that overall 5-year survival rate was 77.3 % for the well-differentiated type and 76.7 % for the moderately differentiated type, which was not statistically significant [6]. In this study, histologic differentiation did not have a significant impact on survival rate by the log rank test results.

## Conclusions

These results suggest that good survival rate can be obtained with surgical treatment only, and stage of oral cancer, cervical lymph node metastasis and stage, recurrence or metastasis, time of recurrence, and metastasis have a significant effect on survival rate in oral cancer patients.

### Consent

The authors declare that they have no competing interests.

## Abbreviations

ACC: adenoid cystic carcinoma
ATFAR: alive; tumor-free; after recurrence
ATFNR: alive; tumor-free; no recurrence
AWPRM: alive with persistent/recurrent/metastatic disease
BCC: basal cell carcinoma
DPO: dead; postoperative
DSS: disease specific 5-year survival rate
DTF: dead; tumor-free
DWC: dead; with cancer (primary/recurrent/metastatic)
GCC: ghost cell carcinoma
MC: mucoepidernoid carcinoma
Meta: metastasis
MM: malignant melanoma
Recur: recurrence
Sar: sarcoma
SCC: squamous cell carcinoma
VC: verrucous carcinoma
